# Predictors of Treatment Failure and Second-Line Therapy Outcomes After Rezūm Therapy for Benign Prostatic Hyperplasia

**DOI:** 10.7759/cureus.112699

**Published:** 2026-07-15

**Authors:** Mohamed Alhabib, Philipp Engert, Armin Soave, Florian Wagenlehner, Martin Friedrich, Khaled Alghueiri

**Affiliations:** 1 Pediatric Urology, Clinic for Urology, Helios Klinikum Krefeld, Krefeld, DEU; 2 Clinic for Urology, Universitätsklinikum Hamburg-Eppendorf, Hamburg, DEU; 3 Pediatric Urology and Andrology, Clinic for Urology, Justus-Liebig-Universität Gießen, Giessen, DEU

**Keywords:** benign prostatic hyperplasia, lower urinary tract symptoms, postvoid residual volume, reintervention, rezūm, treatment failure

## Abstract

Background

Rezūm water vapor thermal therapy has emerged as a minimally invasive treatment option for benign prostatic hyperplasia (BPH). However, data regarding predictors of treatment failure and outcomes of second-line therapies after failed Rezūm therapy remain limited. This study aimed to evaluate functional outcomes, predictors of treatment failure, and outcomes of second-line therapies following Rezūm therapy.

Methods

We conducted a retrospective, single-center study of patients who underwent Rezūm therapy between January 2019 and January 2023. Baseline demographic and clinical characteristics, including age, prostate volume, prostate-specific antigen (PSA), post-void residual volume (PVR), and International Prostate Symptom Score (IPSS), were collected. Treatment failure was defined as the need for secondary intervention. Multivariable logistic regression was performed to identify predictors of treatment failure. Outcomes of second-line therapies and incidental prostate cancer (iPCa) detection were analyzed.

Results

A total of 106 patients were included in the final analysis, with a median follow-up of 34 months. The median IPSS reduction after Rezūm was 6 points. Overall, postoperative complications occurred in 28 of 106 patients (26.4%). During the follow-up period, 33 of 106 patients (31.1%) required a secondary intervention. Elevated baseline PVR was identified as an independent predictor of treatment failure (OR 1.009, 95% CI 1.004-1.013; p < 0.001), whereas age, prostate volume, and baseline IPSS were not significantly associated with treatment failure. Among patients requiring secondary intervention, patients undergoing ablative retreatment experienced significant symptom improvement, with a mean IPSS reduction of 10.5 points (95% CI 8.9-12.1; p < 0.001). iPCa was detected in 3 of 28 patients (10.7%) undergoing histological evaluation after secondary surgery.

Conclusions

Rezūm therapy was associated with significant symptom improvement in patients with symptomatic BPH. Elevated baseline PVR was independently associated with an increased likelihood of treatment failure. Patients undergoing ablative retreatment experienced substantial postoperative symptom improvement following failed Rezūm therapy. Careful patient selection and assessment of bladder dysfunction may improve long-term treatment durability.

## Introduction

Benign prostatic hyperplasia (BPH) is one of the most common urological diseases among aging men and frequently results in lower urinary tract symptoms (LUTS), impaired quality of life, and progressive bladder outlet obstruction [1,2]. The prevalence of BPH increases substantially with age and affects the majority of men over the age of 60 years [3]. Furthermore, disease progression may result in recurrent urinary tract infections, urinary retention, or renal insufficiency in advanced cases [4]. Consequently, the management of symptomatic BPH remains an important component of daily urological practice.

Medical therapy represents the standard first-line treatment for patients with moderate LUTS [5]. However, a substantial proportion of patients experience inadequate symptom relief, medication intolerance, or progressive disease requiring surgical intervention. According to the European Association of Urology (EAU) guidelines, transurethral resection of the prostate (TUR-P) remains the standard surgical treatment for men with prostate volumes of 30-80 mL, whereas laser enucleation procedures such as holmium laser enucleation of the prostate (HoLEP) are recommended for larger prostates because of their superior efficacy and durability [6]. Nevertheless, these procedures remain associated with perioperative morbidity and potential adverse effects on ejaculatory function [7]. These limitations have contributed to increasing interest in minimally invasive surgical therapies (MISTs), particularly among elderly and comorbid patients seeking less invasive treatment alternatives.

Rezūm water vapor thermal therapy is a minimally invasive treatment modality utilizing convective radiofrequency-generated water vapor energy to induce targeted thermal ablation of prostatic tissue [8]. The procedure can be performed under local anesthesia or sedation and has gained increasing popularity because of its favorable safety profile and preservation of sexual function [9]. Previous studies demonstrated significant improvements in International Prostate Symptom Score (IPSS), quality of life, urinary flow parameters, and post-void residual volume (PVR) following Rezūm therapy [10]. Long-term data from randomized controlled trials further demonstrated durable symptom improvement and relatively low secondary intervention rates over follow-up periods of up to five years [11]. Consequently, Rezūm therapy has emerged as an increasingly utilized treatment option in routine clinical practice for patients with symptomatic BPH.

Despite favorable functional outcomes after Rezūm therapy, treatment failure remains a clinically relevant challenge. Treatment failure was defined as the need for second-line surgical intervention, as persistent or recurrent LUTS may continue to be managed conservatively or medically without necessarily indicating failure of the initial procedure. Surgical retreatment was therefore selected as a clinically robust endpoint reflecting failure of the initial treatment. Although previous studies have reported secondary intervention rates after Rezūm therapy, data regarding predictors of treatment failure remain limited [12]. Furthermore, evidence regarding outcomes following second-line therapy after failed Rezūm treatment remains scarce. In particular, real-world studies evaluating second-line treatment strategies and their outcomes following unsuccessful Rezūm therapy are lacking [13].

Therefore, the primary objective of the present study was to identify predictors of treatment failure following Rezūm therapy. Secondary objectives were to evaluate functional outcomes, the outcomes of second-line therapy after Rezūm therapy, and the incidence of incidental prostate cancer (iPCa) detected during secondary surgical intervention, as histopathological assessment becomes available only after tissue-removing salvage procedures. This outcome was further evaluated to determine whether the incidence of iPCa following secondary surgery after Rezūm therapy is comparable to the rates reported after primary surgical treatment for BPH.

## Materials and methods

Patients, setting, and study approval

**Figure 1:**
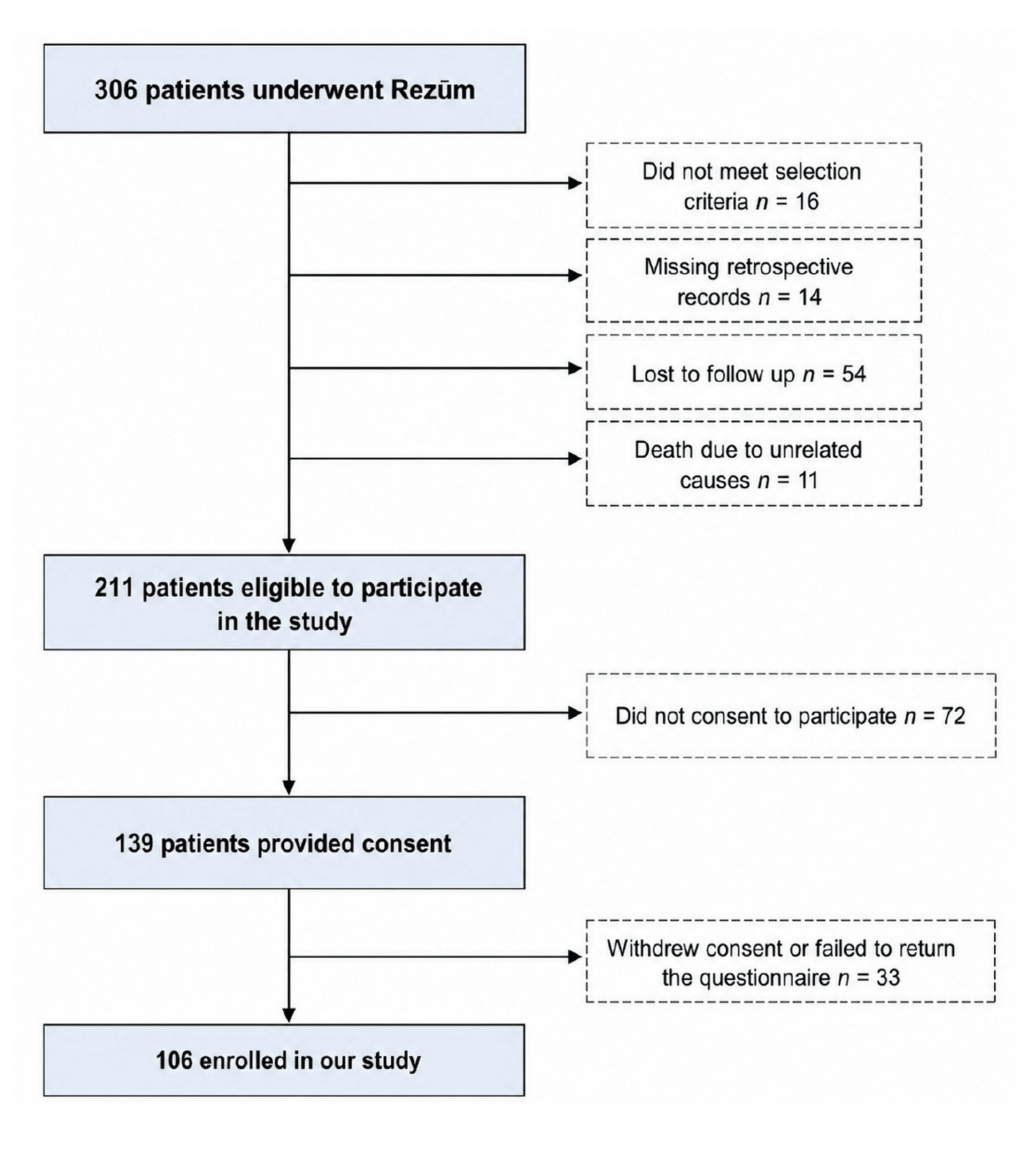
Study enrollment and follow-up Flowchart illustrating patient selection and enrollment. Of 306 patients who underwent Rezūm therapy between January 2019 and January 2023, 95 were excluded because they did not meet selection criteria (n = 16), had missing retrospective records (n = 14), were lost to follow-up (n = 54), or died from unrelated causes (n = 11). Of the remaining 211 eligible patients, 72 declined participation. A total of 139 patients provided consent, of whom 33 subsequently withdrew consent or failed to return the study questionnaire. The final study cohort consisted of 106 patients.

We conducted a retrospective single-center study at the Department of Urology, Helios Hospital Krefeld, Krefeld, Germany, involving 306 patients who underwent Rezūm therapy between January 2019 and January 2023. The reduction from the initial cohort to the final study population resulted from predefined exclusion criteria. Patients who could not be contacted because of changes in their address were classified as lost to follow-up (n = 54), whereas patients who declined participation (n = 72) and patients who initially agreed to participate but subsequently withdrew informed consent (n = 33) were also excluded, as detailed in the flow chart (Figure 1). 

The inclusion criteria were men aged ≥50 years with symptomatic BPH, defined as an IPSS ≥8 and a prostate volume of at least 40 mL, who had failed medical therapy with alpha-1 adrenergic receptor antagonists and/or 5-alpha reductase inhibitors for at least six months. Patients were required to have a minimum follow-up of 12 months after Rezūm. The exclusion criteria included patients with prostate cancer, previous prostate surgeries, or incomplete retrospective medical records.

Before Rezūm, all patients underwent a preoperative evaluation, including a digital rectal examination (DRE) and prostate-specific antigen (PSA) screening based on the EAU guidelines. Patients with abnormal PSA levels or abnormal DRE results underwent prostate biopsy or magnetic resonance imaging. Baseline clinical data were collected retrospectively from electronic medical records, whereas postoperative outcomes were assessed using a standardized follow-up questionnaire administered once after a minimum follow-up of 12 months and supplemented by review of the medical records, where applicable. The study received approval from the Ethics Committee of the Medical Association of North Rhine, Approval No. 2023201. All participants provided written informed consent after receiving information about the study’s objectives and potential risks and benefits.

Data collection and study design

Baseline demographic and clinical data, including prostate volume, age, PSA, PVR volume, and initial IPSS scores, were retrospectively collected from medical records at the time of the procedure. The IPSS is a validated questionnaire consisting of seven symptom questions, each scored from 0 to 5, yielding a total score ranging from 0 to 35, with scores of 0-7 indicating mild, 8-19 moderate, and 20-35 severe LUTS [14].

To assess treatment outcomes, a single standardized follow-up questionnaire was administered once during the study period to all eligible participants after a minimum follow-up of 12 months. The questionnaire collected updated IPSS scores, postoperative complications, details of any second-line therapies, and, where applicable, histopathological findings. All participants completed the same follow-up questionnaire. If patients underwent a second intervention, follow-up was performed at least 12 months after the secondary procedure to collect data on the outcomes of the second intervention.

Rezūm procedure

All Rezūm procedures were performed by two experienced consultant urologists using the Rezūm System (Boston Scientific, Marlborough, MA, USA) according to the manufacturer’s recommended treatment protocol. The procedures were performed under either general or spinal anesthesia with the patient in the lithotomy position. The number and location of water vapor injections were determined according to prostate anatomy and size, with sequential injections administered approximately every 1 cm along the right and left lateral lobes from the bladder neck to the proximal verumontanum. Median lobe enlargement, when present, was routinely treated. At the end of the procedure, a transurethral Foley catheter was inserted in all patients and routinely maintained for one week before removal.

Statistical analysis

Continuous variables were summarized as means with standard deviations (SDs) or medians with interquartile ranges (IQRs), as appropriate according to their distribution, while categorical variables were presented as frequencies and percentages. Normality of continuous variables was assessed using the Shapiro-Wilk test. Changes in IPSS before and after Rezūm therapy were evaluated using a paired-samples t-test. Correlations between continuous variables were assessed using Pearson correlation analysis. Linear regression analysis was performed to identify predictors of IPSS reduction. Analyses evaluating IPSS reduction were restricted to patients with available paired preoperative and postoperative IPSS data (n = 71), as IPSS reduction could only be calculated in patients with complete paired measurements. No imputation of missing data was performed. To identify predictors of treatment failure, univariate logistic regression analyses were initially performed, followed by a multivariable logistic regression model including age, prostate volume, baseline IPSS, and PVR as covariates. Statistical significance was defined as a two-sided p-value < 0.05. All statistical analyses were performed using R (version 4.4; R Foundation for Statistical Computing, Vienna, Austria) and jamovi (version 2.6; The jamovi Project, Sydney, Australia).

## Results

Patient demographics and outcome measures

A total of 106 patients were included in the final analysis. Minimum follow-up time after initial Rezūm therapy was 12 months. The median follow-up duration was 34 months. Patient characteristics and postoperative outcomes are summarized in Table 1. The postoperative IPSS assessed at least 12 months after the initial Rezūm therapy decreased from a median (IQR) of 19 (15-25) preoperatively to 9.5 (5-16) postoperatively, corresponding to a median IPSS reduction of 6 (2-10) points. Paired-samples t-test confirmed a statistically significant improvement in IPSS after Rezūm therapy (Table 2). The results of the Pearson correlation analysis and linear regression analysis are presented in Tables 3-4.

**Table 1:** Baseline characteristics and outcome measures after Rezūm (n = 106) Data are presented as median (IQR), as appropriate. PVR: postvoid residual volume; PSA: prostate-specific antigen; IPSS: International Prostate Symptom Score; IQR: interquartile range

Variable	Median (IQR)
Age, years	67 (59-74)
Prostate volume (mL)	48 (30-60)
PVR (mL)	100 (35-165)
PSA pre (ng/mL)	2.5 (2.0-5.9)
Preoperative IPSS	19 (15-25)
Postoperative IPSS	9.5 (5-16)
IPSS reduction	6 (2-10)

**Table 2:** Paired-samples t-test comparing preoperative and postoperative IPSS after Rezūm therapy Paired Student’s t-test comparing preoperative and postoperative IPSS. Analysis was performed using n = 71 patients with available postoperative IPSS data. IPSS reduction was calculated as the difference between preoperative and postoperative IPSS scores. IPSS: International Prostate Symptom Score

Outcome	Mean Difference	t	Cohen's d	p-value
IPSS reduction	7.06	7.44	0.889	<0.001

**Table 3:** Pearson correlation analysis of factors associated with IPSS reduction after Rezūm therapy Pearson correlation analysis evaluating the association between baseline clinical variables and IPSS reduction after Rezūm therapy. Analysis was performed using n = 71 patients with available postoperative IPSS data. IPSS: International Prostate Symptom Score; PSA: prostate-specific antigen (ng/mL); PVR: postvoid residual volume (mL)

Variable	Pearson's r	p-value
Age	0.009	0.943
Reintervention	-0.106	0.380
Baseline PSA	0.102	0.397
PVR	0.294	0.013
Prostate volume	0.247	0.038

**Table 4:** Linear regression analysis of predictors of IPSS reduction after Rezūm therapy Multivariable linear regression analysis evaluating independent predictors of IPSS reduction after Rezūm therapy. Analysis was performed using n = 71 patients with available preoperative and postoperative IPSS data. Model fit: R² = 0.163, adjusted R² = 0.112, F(4,66) = 3.22, p = 0.018. β: unstandardized regression coefficient; SE: standard error; CI: 95% confidence interval; IPSS: International Prostate Symptom Score; PSA: prostate-specific antigen (ng/mL); PVR: postvoid residual volume (mL)

Predictor	β	SE	95% CI	t	p-value
Intercept	3.958	6.773	-9.565 to 17.481	0.584	0.561
Age	-0.070	0.103	-0.276 to 0.136	-0.679	0.499
PVR	0.029	0.010	0.008 to 0.049	2.810	0.007
Baseline PSA	0.062	0.275	-0.488 to 0.612	0.224	0.824
Prostate volume	0.091	0.041	0.009 to 0.172	2.223	0.030

Retrograde ejaculation occurred in 19 patients (17.9%). Out of the 106 patients, 28 complications were reported only in the first three months after Rezūm therapy, corresponding to an overall complication rate of 26.4%. Complications were classified according to the Clavien-Dindo system [15]. The detailed breakdown of adverse events is presented in Table 5.

**Table 5:** Complications according to Clavien-Dindo classification (n = 106) Postoperative complications following Rezūm therapy according to the Clavien-Dindo classification. Data are presented as the number of events and affected patients (%).

Clavien-Dindo grade	Adverse event	Events (n)	Patients n (%)
Grade I	Mild pain	7	6.6
Grade II	Urinary retention (requiring catheter)	15	14.1
Grade II	Prostatitis / Cystitis	6	5.5
Total		28	26.4

Predictors of Rezūm failure

During the follow-up period (median 34 months), 33 of 106 patients (31.1%) required a secondary intervention. Multivariable logistic regression analysis identified elevated PVR volume as an independent predictor of treatment failure after Rezūm therapy (OR 1.009, 95% CI 1.004-1.013; p < 0.001). In contrast, age, prostate volume, and baseline IPSS were not significantly associated with reintervention after Rezūm therapy. Detailed results of the univariate and multivariable analyses are presented in Table 6.

**Table 6:** Univariate and multivariable logistic regression analysis of predictors of treatment failure after Rezūm therapy (n = 106) Univariate and multivariable logistic regression analyses were performed to identify predictors of treatment failure after Rezūm therapy. Odds ratios (ORs) with 95% confidence intervals (CIs) are presented. Variables included in the multivariable model were age, prostate volume, postvoid residual (PVR) volume, and preoperative International Prostate Symptom Score (IPSS). Statistical significance was defined as p < 0.05.

Variable	Univariate OR (95% CI)	p-value	Multivariable OR (95% CI)	p-value
Age, years	0.994 (0.948-1.040)	0.804	1.003 (0.942-1.067)	0.932
Prostate volume (mL)	0.988 (0.970-1.010)	0.181	0.996 (0.975-1.018)	0.721
PVR (mL)	1.009 (1.005-1.013)	<0.001	1.009 (1.005-1.014)	<0.001
Preoperative IPSS	1.038 (0.976-1.104)	0.232	1.014 (0.935-1.100)	0.732

Second-line therapy outcomes

**Figure 2:**
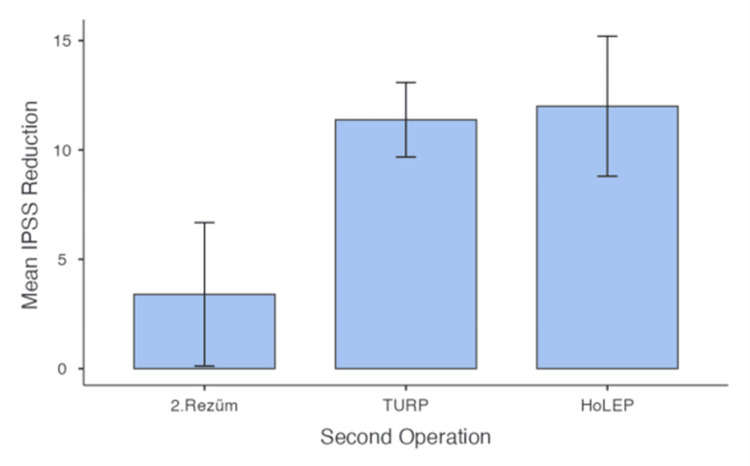
IPSS reduction after salvage interventions (n = 32) Mean reduction in IPSS following second-line therapy after Rezūm failure. Bars represent the mean IPSS reduction, and error bars indicate 95% confidence intervals. Patients undergoing TURP and HoLEP demonstrated greater symptom improvement than those undergoing repeat Rezūm therapy. One patient treated with PAE was excluded from the figure because a meaningful graphical comparison was not possible. IPSS: International Prostate Symptom Score; TURP: transurethral resection of the prostate; HoLEP: holmium laser enucleation of the prostate; PAE: prostatic artery embolization

Among the 33 patients who underwent a second operation, 21 (63.6%) underwent TUR-P, seven (21.2%) underwent HoLEP, four (12.1%) underwent a second Rezūm procedure, and one patient underwent prostatic artery embolization (PAE). Furthermore, postoperative IPSS scores were evaluated at least 12 months after second-line therapy (Figure 2). Patients who underwent ablative second-line interventions experienced significant improvement in IPSS, with a mean reduction of 10.5 points (95% CI 8.9-12.1; p < 0.001). Mean IPSS reduction was 10.45 after TURP, 10.67 after HoLEP, 2.00 after repeat Rezūm therapy, and 0 after PAE.

Incidence of iPCa following second-line therapy after Rezūm

Regarding the histological evaluation, 10.7% (3 of 28) of patients who underwent histological evaluation had iPCa. Two had a Gleason score of 3 + 3, and one had a Gleason score of 4 + 3. The mean age (SD) of this group was 70.2 (5.7) years, and the median (IQR) PSA was 3.6 ng/mL (2.02-5.9).

## Discussion

The management of BPH has progressively shifted towards minimally invasive treatment strategies aiming to reduce perioperative morbidity while preserving functional outcomes and sexual function [16]. In recent years, Rezūm water vapor thermal therapy has emerged as an increasingly utilized minimally invasive option for patients with symptomatic BPH. In the present study, Rezūm therapy was associated with a median IPSS reduction of 6 points after the initial intervention. Similar postoperative symptom improvement has previously been reported in prospective and real-world Rezūm studies. Previous studies have likewise demonstrated durable postoperative improvement in LUTS and quality of life following Rezūm therapy [17].

Patients with higher baseline PVR volumes were more likely to experience treatment failure following Rezūm therapy. This association may reflect underlying detrusor underactivity secondary to prolonged bladder outlet obstruction, resulting in impaired bladder emptying and persistent residual urine despite initial symptom improvement. Similar associations between baseline bladder dysfunction, impaired emptying, and less durable postoperative outcomes have previously been reported in patients undergoing minimally invasive BPH therapies [18,19]. During the follow-up period (median 34 months), 33 of 106 patients (31.1%) required a secondary intervention. Reported retreatment rates after Rezūm therapy in the current literature are generally lower, particularly in randomized controlled trials and highly selected patient cohorts [20,21]. The higher retreatment rate observed in our cohort may reflect differences in real-world patient selection, baseline disease severity, longer follow-up, and the inclusion of patients with more advanced bladder outlet obstruction and elevated baseline PVR volumes.

Furthermore, we found that ablative interventions such as TURP and HoLEP represented the most common second-line therapies after Rezūm failure. Patients undergoing TURP and HoLEP experienced significant postoperative symptom improvement following retreatment. Although these findings are consistent with the durable symptomatic relief reported after ablative procedures for persistent bladder outlet obstruction [22,23], the relatively small subgroup sizes and observational design of the present study preclude direct comparisons between different salvage treatment modalities. Repeat Rezūm therapy was performed less frequently in our cohort, possibly reflecting the preference for more definitive desobstructive procedures after initial therapy failure. These findings suggest that secondary intervention after Rezūm therapy should not necessarily be interpreted solely as treatment failure, but may instead represent part of an individualized therapeutic strategy for selected patients with persistent or recurrent bladder outlet obstruction.

Our findings are consistent with those of our previous study evaluating treatment failure after PAE [24], which similarly identified elevated PVR as an independent predictor of treatment failure and reported greater symptom improvement following ablative secondary surgery than repeat minimally invasive interventions. In contrast, whereas both baseline PVR and IPSS independently predicted treatment failure in the previous PAE study, only elevated baseline PVR remained independently associated with treatment failure following Rezūm therapy.

In this study, we observed an iPCa rate of 10.7% following secondary BPH surgeries after Rezūm therapy. Similar rates of iPCa after surgery for presumed BPH have previously been described in contemporary studies despite widespread PSA screening and modern imaging techniques [25]. Although only three cases were identified, the observed incidence was comparable to previously reported series. These findings suggest that previously undiagnosed prostate cancer may contribute to persistent LUTS in a small subset of patients undergoing secondary surgery after Rezūm therapy; however, this observation should be considered exploratory.

We acknowledge several limitations in our study. The retrospective single-center design may have introduced selection bias and may limit the generalizability of our findings. In addition, the substantial reduction from the initial cohort to the final study population, resulting from losses to follow-up, declining participation, and withdrawal of informed consent, may have introduced response bias and affected the representativeness of the analyzed cohort. Postoperative follow-up relied partly on patient-reported questionnaire assessments, while objective postoperative parameters such as uroflowmetry, repeated PVR measurements, and bladder function assessments were not consistently available, limiting the correlation between subjective symptom improvement and objective functional outcomes. Furthermore, important clinical variables, including prostate morphology, bladder contractility, urinary flow rate (Qmax), catheter dependence, ongoing medical therapy, and selected comorbidities, were not incorporated into the multivariable analysis, and residual confounding therefore cannot be excluded. Finally, the relatively small sample sizes of the repeat Rezūm and PAE subgroups limit definitive comparisons between second-line treatment modalities, and these findings should therefore be interpreted cautiously.

Future prospective multicenter studies with larger cohorts and standardized long-term follow-up protocols integrating symptom assessment with objective functional and imaging measures are needed to better identify predictors of treatment failure after Rezūm therapy. Improved characterization of bladder dysfunction, obstruction severity, prostate morphology, and patient selection may further optimize long-term treatment durability and postoperative outcomes following minimally invasive BPH therapies.

## Conclusions

Rezūm therapy was associated with significant postoperative symptom improvement and represents a minimally invasive treatment option for patients with symptomatic BPH. However, treatment failure remains clinically relevant, with elevated baseline PVR being independently associated with an increased likelihood of secondary intervention. Patients undergoing TURP and HoLEP after Rezūm failure experienced substantial postoperative symptom improvement; however, these findings should be interpreted cautiously given the observational design and limited subgroup sizes. Careful patient selection and preoperative counseling remain essential to optimize long-term outcomes after minimally invasive therapy.
